# Cross-sectional study of approaches to diagnosis and management of dogs with immune-mediated haemolytic anaemia in primary care and referral veterinary practices in the United Kingdom

**DOI:** 10.1371/journal.pone.0257700

**Published:** 2021-09-20

**Authors:** James W. Swann, Sarah Tayler, Harriet Hall, Richard Sparrow, Barbara J. Skelly, Barbara Glanemann

**Affiliations:** 1 Columbia Stem Cell Initiative, Columbia University, New York, NY, United States of America; 2 Department of Clinical Science and Services, Royal Veterinary College, Hatfield, United Kingdom; 3 Department of Veterinary Medicine, University of Cambridge, Cambridge, United Kingdom; University of Lincoln, UNITED KINGDOM

## Abstract

**Objectives:**

To determine whether veterinarians in primary care practices (PCPs) and board-certified clinicians (BCCs) approach treatment of dogs with immune-mediated haemolytic anaemia (IMHA) similarly, and whether practitioners with more experience treat similarly to those with less experience. We hypothesised those in PCPs would show more variation in their approach to similar cases than BCCs.

**Methods:**

A cross-sectional study was conducted by distributing a questionnaire to BCCs and veterinarians in PCPs. The questionnaire included direct questions and a number of clinical scenarios intended to capture approaches to common treatment problems.

**Results:**

Questionnaire responses were received from 241 veterinarians, including 216 in PCPs and 25 BCCs. Veterinarians in both settings used similar tests for diagnosis of IMHA, but BCCs performed more tests to exclude underlying causes of ‘associative’ disease. All veterinarians reported use of similar initial dosages of glucocorticoids (median 2 mg/kg per day in both groups, *p* = 0.92) but those used by more experienced practitioners were higher than those with less experience. Most veterinarians made allowances for the weight of dogs, using lower prednisolone dosages in a clinical scenario involving a 40 kg dog compared to a 9 kg dog (*p* = 0.025 for PCP, *p* = 0.002 for BCC). BCCs reported greater use of combinations of immunosuppressive drugs (*p*<0.0001) and of antithrombotic drugs (*p*<0.0001); use of antithrombotic drugs was also less common among more experienced practitioners compared to less experienced.

**Conclusions:**

Approaches to treatment of dogs with IMHA differ between BCCs and those in PCP. These differences may affect design and implementation of future research studies and clinical guidelines.

## Introduction

Immune-mediated haemolytic anaemia (IMHA) is treated commonly in veterinary referral hospitals employing board-certified clinicians (BCCs) [1]. However, most dogs with clinical signs of IMHA are presented initially to a primary care practice (PCP), and many will receive all of their diagnostic investigations and treatment in this setting if referral is unnecessary, unaffordable, or unwanted.

Investigation and management of dogs with IMHA in different locations has the potential to cause important consequences. First, data underpinning all published research studies are derived from referral hospitals, with no information to indicate whether results are representative of, or conclusions applicable to, dogs with IMHA that are never referred. Second, BCCs often lead development of clinical guidelines intended to provide standardised and evidence-based recommendations for investigation and treatment, including the recent American College of Veterinary Internal Medicine (ACVIM) consensus statements on this topic [2,3]. However, implementation of such guidelines may not be feasible in PCPs, particularly if intensive forms of investigation and treatment are recommended in all cases. Thirdly, because most BCCs pass through comparable programmes of specialisation, referral hospitals are likely to achieve a high level of consistency in their approach to clinical cases, even if unintentionally. In PCPs, even though all veterinarians historically have trained in specialist veterinary school hospitals under the supervisions of BCCs, we suspect investigation and treatment of the same disease could be more varied, dependent on the experience and interests of attending veterinarians. Finally, the areas identified by veterinarians as deserving of further investigation differ, with a large proportion of specialists desiring clinical studies on thromboprophylaxis in IMHA [4], whereas, in our experience, veterinarians in PCP more often consider the merits of long-term immunosuppressive treatment and risks of vaccination.

Two recent ACVIM consensus statements have provided recommendations for diagnosis and treatment of IMHA in dogs, providing a classification system for confidence of diagnosis as ‘diagnostic’, ‘supportive’, or ‘suspicious’ according to the number of features of immune-mediated red blood cell destruction and haemolysis that are detectable in each case.

Building on these observations, assumptions, and new resources, the objective of our work was to gain information on current approaches to investigation and treatment of IMHA outside referral hospitals, hypothesising that approaches would be more varied in PCPs compared to referral practices and would differ with the experience of veterinarians. To achieve this, we designed a questionnaire that was distributed among veterinarians in primary and specialist practices in the United Kingdom (UK), incorporating a number of clinical scenarios intended to capture differences in clinical approach. Importantly, this work was not intended to be judgemental of veterinarians working in PCPs but to generate data to inform future research and clinical governance projects of the differences in management of IMHA in different settings.

## Materials and methods

### Study design

A cross-sectional study was conducted by distributing a single-source written questionnaire to veterinarians in PCP and to BCCs in the UK between January 2016 and October 2017. All participants gave written informed consent for participation in the study, and the questionnaire and study protocol were approved by the Clinical Research Ethical Review Board of the Royal Veterinary College (reference number: URN2015_1389).

### Questionnaire

We designed the questionnaire to capture information on topics relating to the diagnosis and treatment of IMHA in PCP and referral practices based on our experience of treating this disease and interacting with owners and other veterinarians. In addition, we designed a number of simple clinical scenarios that reflected common problems or differences in opinion that we have encountered. Veterinarians with recent experience of PCP in our institutions were asked to complete the questionnaire and provide feedback, which was used to modify several questions before a final version was created using online software (SurveyMonkey, www.surveymonkey.com), in which participants could navigate forwards and backwards through the survey. Ethical approval for distribution of the questionnaire was granted by an institutional ethical review board (URN2015_1389). A transcript of the questionnaire is available in **S1 File**.

### Distribution to PCPs

A link to the questionnaire was sent in an e-mail to 1,637 veterinarians and 180 practices who were members of a mailing list previously maintained by a university referral hospital (Royal Veterinary College) in January 2016. In addition, we obtained publicly-available e-mail addresses for PCPs from a national database (register of the Royal College of Veterinary Surgeons, findavet.rcvs.org.uk), from which we randomly selected 549 practices in 3 different geographical regions and sent an e-mail with an explanation of the study and the link to the questionnaire. Owing to the manner in which the questionnaire was distributed, it was not possible to calculate a definite response rate, but 2,366 separate e-mails were sent to individuals or practices during the course of the study, of which 160 were undeliverable. Distribution of the questionnaire was completed before the implementation of Regulation (EU) 2016/679 (General Data Protection Regulation) [5] that would not permit these activities today.

### Distribution to BCCs

The names of all American or European board-certified specialists in internal medicine and emergency and critical care practicing in the United Kingdom (UK) were obtained from central databases (VetSpecialists, www.vetspecialists.com; ECVIM listings, www.ecvim-ca.org/specialist-listings). An e-mail was sent to each of these individuals in August 2017, or to their practice if a personal e-mail address was not found, providing an explanation of the study and a link to the questionnaire. In total, e-mails were sent to 69 internal medicine specialists and 8 emergency and critical care specialists.

### Data analysis

Questionnaire responses were copied into spreadsheet software (Excel 2016, Microsoft) and coded for further analysis. Responses from countries other than the UK, which were not solicited, were excluded because availability of tests and drugs and structure of educational programmes differ among countries. Responses from individuals who were not board-certified but working in referral practices were also excluded because their professional role was unknown. Responses were not excluded if some sections were incomplete; the denominator for analysis is provided in relevant sections to indicate how many individuals responded to each question.

In several clinical scenarios, we asked respondents to state the total dose of a drug they would choose to administer. To compare responses, we divided this answer by the stated weight of the dog to derive the dosage. Where we asked respondents to state a new dose of a drug during a dose reduction, we calculated the percentage reduction in dosage from the stated previous dosage.

Statistical analysis was completed using commercially available software packages (SPSS version 22, IBM Corp; Graphpad Prism version 7, Graphpad Software Inc). For continuous variables, distribution was assessed by visual assessment of histograms and using Shapiro-Wilks tests. Because no variables were normally distributed, results were presented as median with inter-quartile range, and groups were compared using Mann-Whitney U tests for independent samples and Wilcoxon Signed rank tests for paired samples. Categorical variables were compared between groups using Chi squared or Fisher’s exact tests, according to the number of cases per cell. The complete dataset is available in **S2 File.**

## Results

### Demographic characteristics

**Fig 1:**
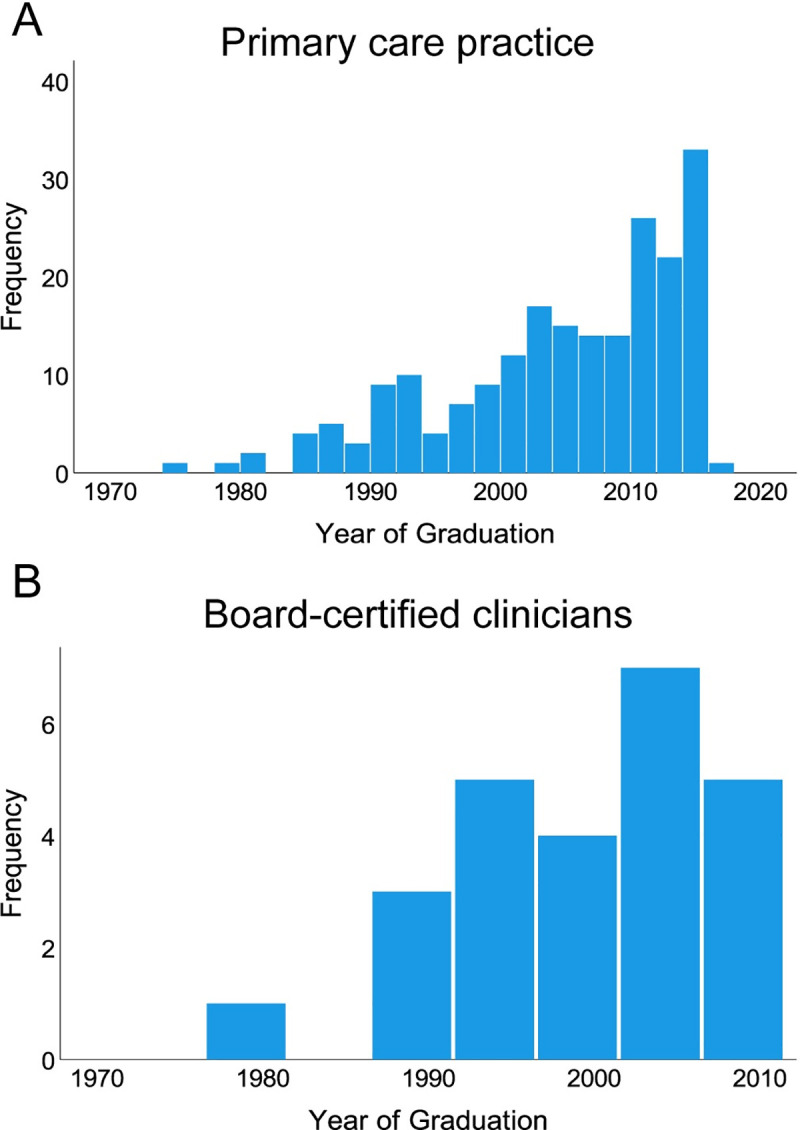
Most respondents graduated after 2000. Histograms showing frequencies of year of graduation for (**A**) veterinarians in primary care practice (PCP), n = 209 and (**B**) board-certified clinicians (BCC), n = 25. Bins represent 2 year periods (**A**) or 5 year periods (**B**).

Questionnaire responses were received from 261 veterinarians, of which 14 were excluded because they were based in a different country. Among the remaining 247 respondents, 187 (75.7%) graduated in the UK, 39 (15.7%) in other European countries, 11 (4.5%) in Australasia, 2 (0.8%) in North America, 1 (0.4%) in Asia, 1 (0.4%) in Africa, with 6 (2.4%) choosing not to provide this information. Among these respondents, 24 were board-certified specialists in internal medicine working in university (n = 8) or private (n = 16) referral practices and one was a board-certified specialist in emergency and critical care working at a private referral practice. Collectively, responses were received from BCCs at 21 different institutions (**Table 1**). There were 6 individuals who were not board-certified but worked in a referral practice; their responses were excluded from further analysis. The remaining 216 respondents worked in PCPs that treated small animals exclusively (n = 170), or a mixture of small and large animals (n = 46). The majority of respondents graduated between 2000 and 2015 regardless of work setting, with distribution shown in **Fig 1**. The majority (158/200, 79.0%) of those in PCP and all BCCs had diagnosed at least one dog with IMHA in the previous year, and those in PCP estimated that a median of 90% (inter-quartile range [IQR]: 75–100) of cases were treated in their practice exclusively without referral.

**Table 1:** Demographic characteristics of questionnaire respondents.

Parameter		Primary care practice	Board-certified clinicians
N		216	25
Gender		170 female (78.7%), 46 male (21.3%)	18 female (72.0%), 7 male (28.0%)
Practice type			
	Small animal only	170	
	Mixed small and large animal	46	
	University referral hospital	3*	8
	Private referral hospital	3*	17
Practice size (full time equivalent veterinary positions, median and IQR)		5.2 (3.0–7.0)	25.0 (18.0–42.0)

IQR: Inter-quartile range.

* Excluded from analysis.

### Diagnosis of IMHA

**Fig 2:**
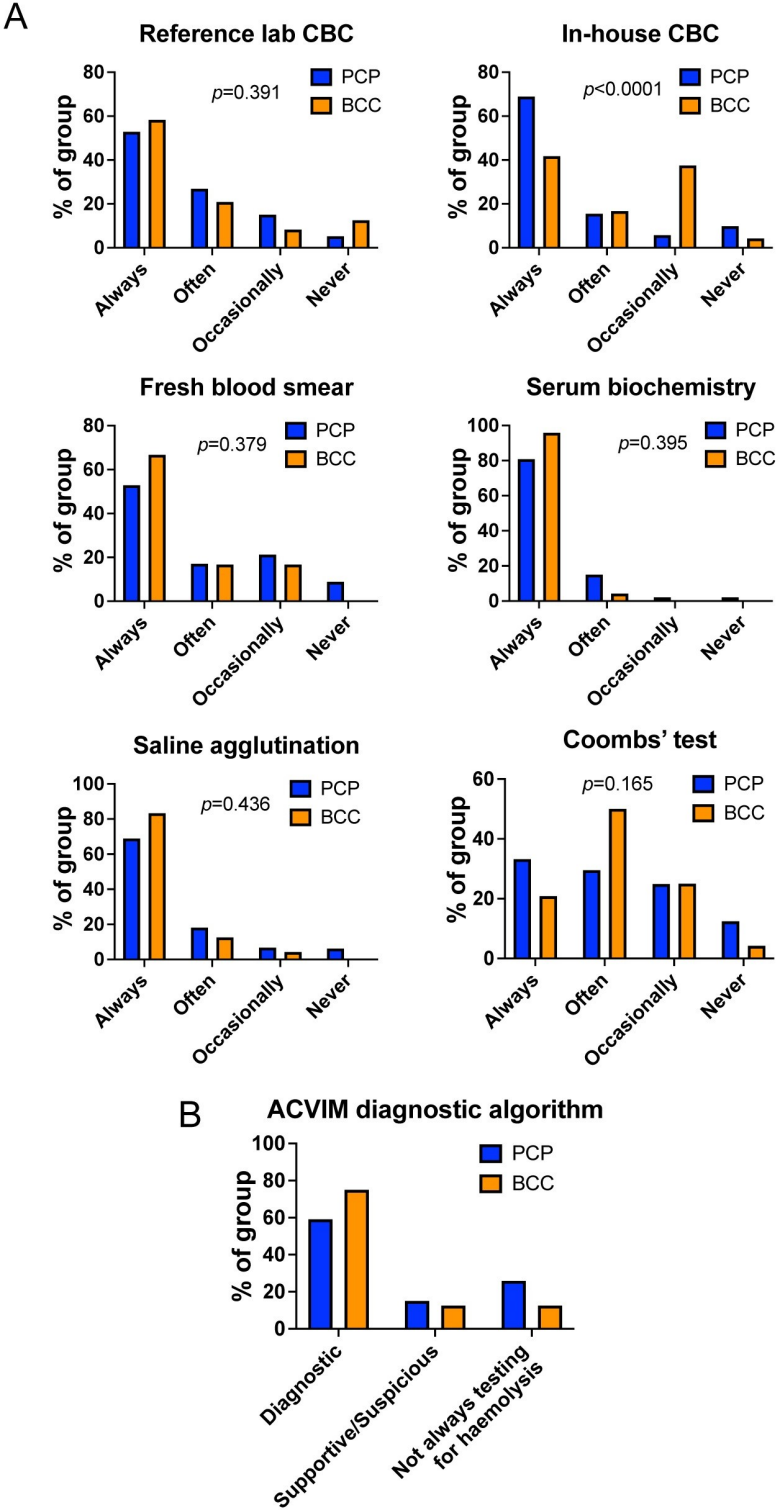
Respondents in primary care and referral practice have a similar approach to diagnosis of IMHA. **(A)** Graphs showing percentage of either board-certified clinicians (BCC, n = 24) or respondents in primary care practice (PCP, n = 193) that reported use of the named tests for diagnosis of IMHA, Chi-squared or Fisher’s exact tests. CBC: complete blood count. **(B)** Graph showing proportion of respondents in each group that always performed sufficient tests for different categories of diagnostic confidence for IMHA as outlined in the ACVIM consensus statement [3].

**Fig 3:**
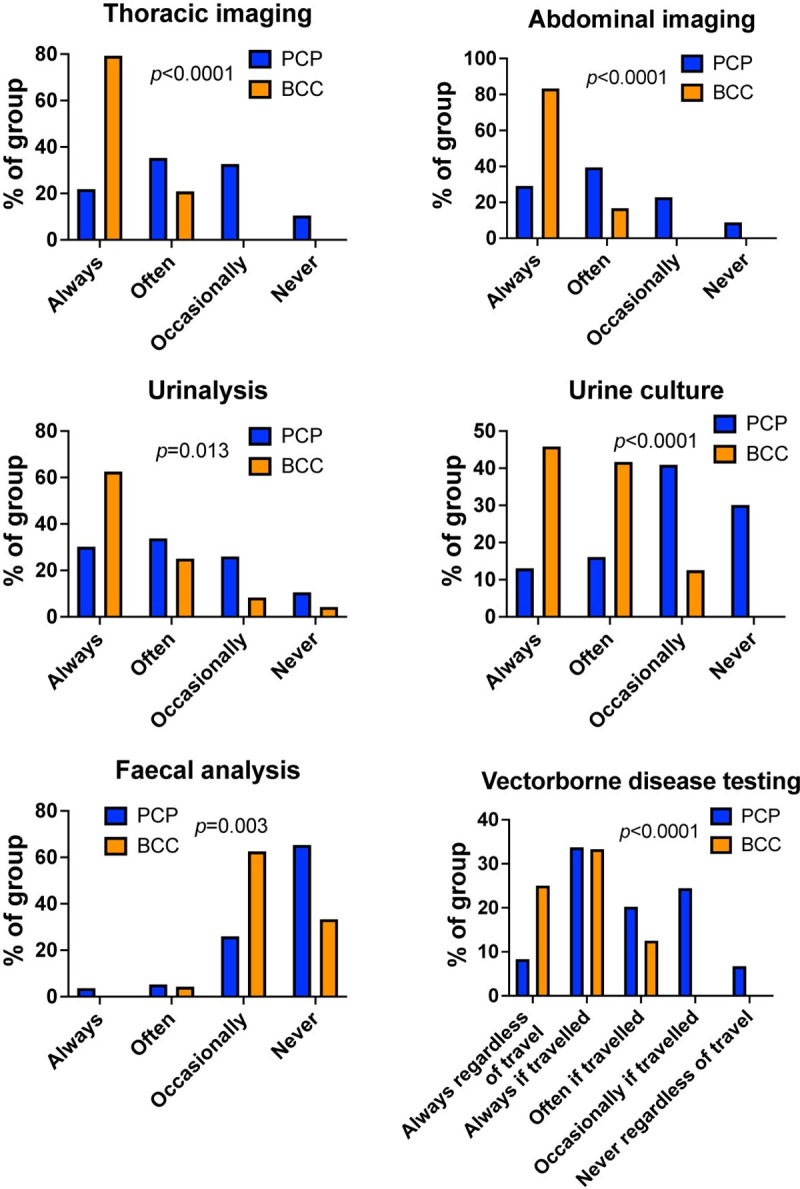
Board-certified clinicians reported more frequent use of additional tests to exclude underlying causes of IMHA. Graphs showing percentage of either board-certified clinicians (BCC, n = 24) or respondents in primary care practice (PCP, n = 193) that reported use of the named tests for diagnosis of IMHA. Chi-squared or Fisher’s exact tests.

When asked which investigations they undertook to reach a diagnosis of IMHA, BCCs and those in PCP commonly performed tests to characterise anaemia, albeit more often in-house at PCPs (complete blood count [CBC] at reference lab, p = 0.391, CBC in-house p<0.0001, blood smear examination in-house, p = 0.379). Respondents from both groups also performed tests at similar rates to establish whether there is concurrent hyperbilirubinaemia suggestive of haemolysis (serum biochemistry, p = 0.395) and to determine whether there are features of immune-mediated red blood cell (RBC) damage (blood smear examination, saline agglutination, p = 0.436, and Coombs’ test, p = 0.165) (**Fig 2A**). To establish whether clinicians were approaching the diagnosis of IMHA in a similar manner to that recommended in the recent ACVIM consensus statement on the same topic [3], we determined whether each respondent always chose to perform sufficient tests for the ‘diagnostic’ category (two tests for potential immune-mediated RBC destruction and one test for haemolysis), for the ‘supportive/suspicious’ category (one test for potential RBC destruction and one test for haemolysis), or whether a test for haemolysis was not always performed, which would preclude a complete diagnosis of IMHA. Doing so, we found most respondents in both groups always performed sufficient tests for the ‘diagnostic’ category, reconciling with the similar patterns of use of individual tests (**Fig 2B**). However, when asking which tests were undertaken to establish if IMHA was associated with an underlying cause, we found BCCs were much more likely to perform additional tests, particularly thoracic and abdominal imaging (both p<0.0001) and urinalysis (p = 0.013). Similarly, although BCCs and those in PCPs performed tests for vectorborne infectious agents at similar rates in those dogs that had travelled outside the UK, BCCs were also more likely to do so in dogs that did not have a history of travel to another country (p<0.0001 for overall comparison, **Fig 3**). Collectively, this suggests BCCs are more concerned about possible underlying causes of IMHA than those in PCPs.

### Transfusion therapy

**Fig 4:**
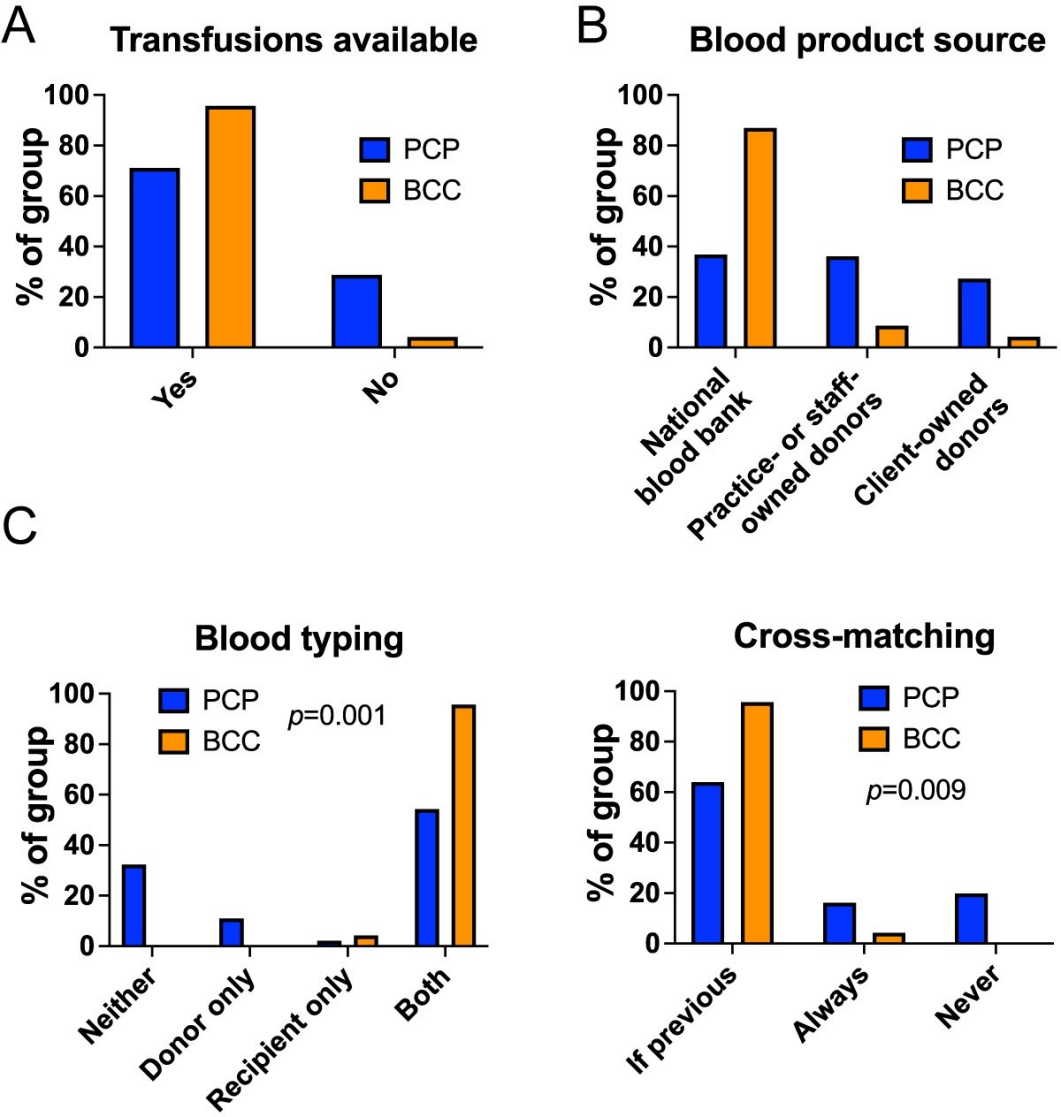
Blood transfusions are widely available in veterinary practice but extent of pre-transfusion testing differs. (**A**) Graph showing proportion of respondents in primary care practice (PCP, n = 191) or of board-certified clinicians (BCC, n = 24) reporting availability of blood transfusions in their practice. (**B**) Graph showing proportion of PCP and BCC respondents reporting indicated sources of blood products in their practice. (**C**) Graphs showing proportion of PCP and BCC reporting use of blood typing (left) and cross-matching (right) in their practices. Chi-squared or Fisher’s exact tests.

The majority of those in both PCPs and specialist practices administered blood transfusions at their own centre (**Fig 4A**), but the source of blood products varied, with most referral practices relying on a national charitable blood bank whereas those in PCPs were more likely to use local donor animals (**Fig 4B**). Almost all BCCs obtained the blood type of donor and recipient before transfusion, but approximately one third of respondents in PCPs did not type either dog (**Fig 4C**, *p* = 0.001 for comparison between groups). Similarly, those in PCPs reported that dogs needing repeated transfusion were less likely to be cross-matched to donor blood before transfusion compared to respondents in specialist practices (**Fig 4C**, *p* = 0.009).

### Immunomodulatory treatment

**Fig 5:**
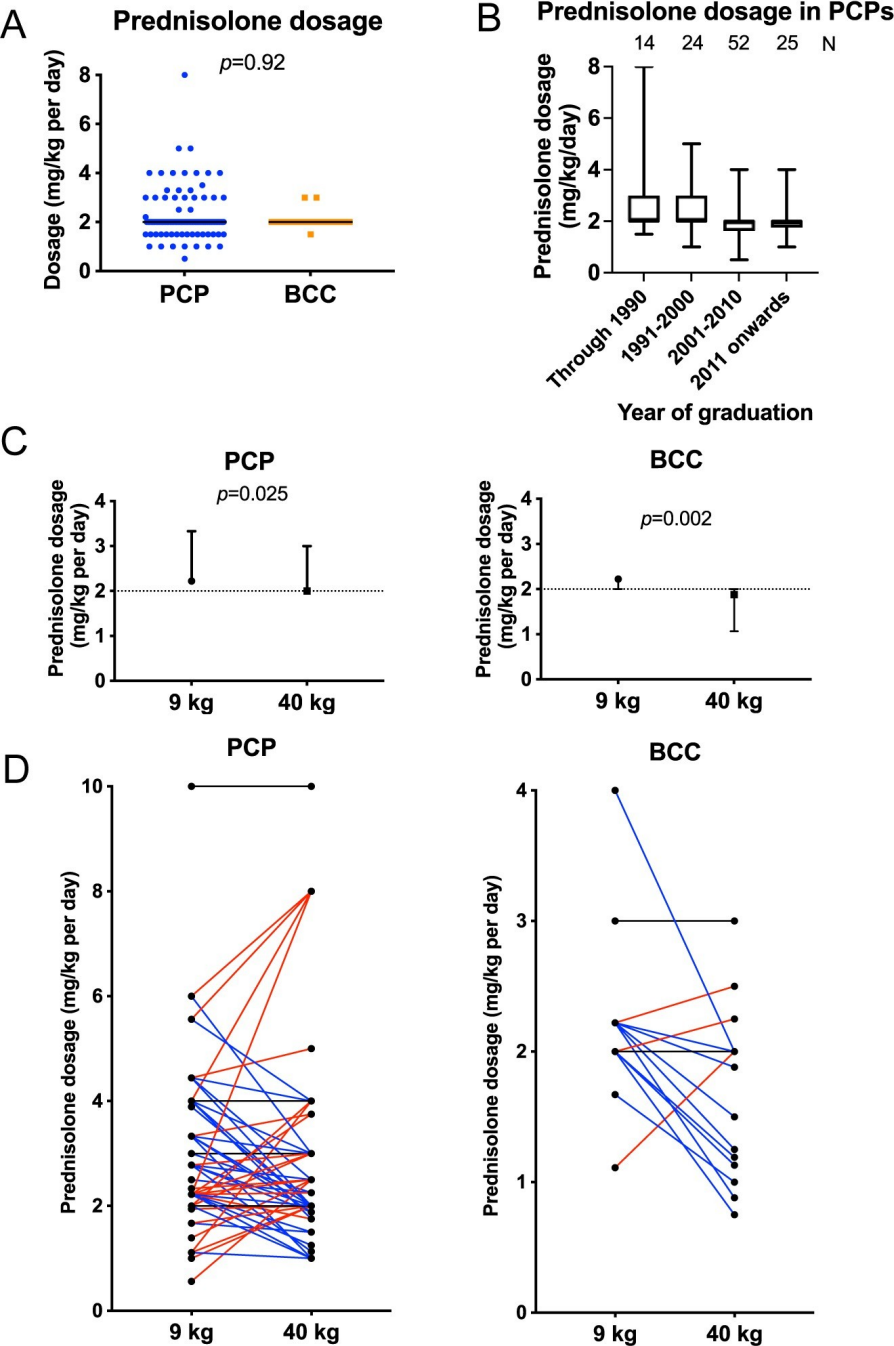
Respondents in primary care practice reported more varied initial dosages of prednisolone. (**A**) Graph showing initial glucocorticoid dosage used by those in primary care practice (PCP, n = 116) or board-certified clinicians (BCC, n = 22). Points represent individual responses, bars represent median. Inter-quartile range values were the same as the median in both groups. Groups compared with Mann-Whitney U test. (**B**) Boxplot showing initial glucocorticoid dosage selected by those in PCP according to year of graduation, annotated with number of respondents per group. Boxes show median with 25^th^ and 75^th^ percentiles, whiskers show minimum and maximum values. (**C**) Graphs showing dosage selected by those in PCP, n = 133 and BCCs, n = 21 in two clinical scenarios concerning initial dosage of glucocorticoids in a dog recently diagnosed with IMHA. Points represent median with inter-quartile range. Dotted lines indicate median value provided in Fig 5A to show difference from answers to that question. Responses compared with Wilcoxon signed rank tests. (**D**) Individual paired responses for those in PCP and BCCs for the two clinical scenarios. Individuals selecting the same dosage are marked with a black line, those using a higher dosage in the heavier dog are marked with a red line, and those using a lower dosage in the heavier dog are marked with a blue line.

Following diagnosis, we asked respondents to state the initial dosage of prednisolone they would administer for treatment of IMHA. An immunosuppressive dosage was selected in all cases, with a clear preference for a dosage of 2.0 mg/kg (median and inter-quartile range values all 2.0 mg/kg) among both BCCs and those working in PCP. There was no difference in dosage between BCCs and those in PCPs (p = 0.905; **Fig 5A**), but the variability in selected dosage was greater in the latter group. Among those in PCP, there was a tendency for those graduating later to use lower initial dosages of prednisolone (**Fig 5B**).

**Fig 6:**
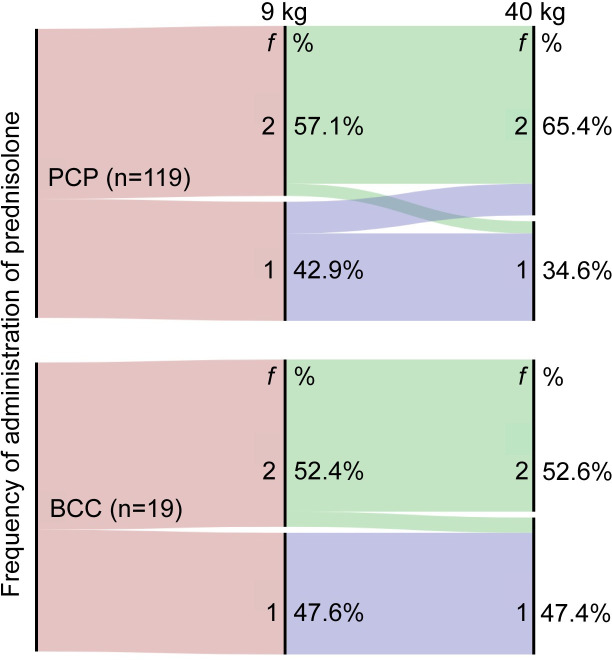
Clinicians administer glucocorticoids once daily or twice daily with similar frequency in clinical scenarios. Alluvial plots showing intended frequency of administration (f) of prednisolone by board-certified clinicians (BCC) (bottom) and those in primary care practices (PCP) (top) in two clinical scenarios when asked to indicate their preferred starting dose of prednisolone for treatment of 2 dogs of different weights, indicated by ‘9 kg’ and ‘40 kg’ over the corresponding nodes. Respondents indicated they administered prednisolone either once daily (‘1’) or twice daily (‘2’) in these clinical scenarios, with some respondents making a different choice between the 2 scenarios.

Because this question represented a simple exercise in recalling an appropriate dosage for treatment of an immune-mediated disease, we explored the topic further by presenting two clinical scenarios, both describing recent diagnosis of IMHA: one in a Dachshund weighing 9 kg and the other in a Rottweiler of 40 kg. In these scenarios, respondents were asked to indicate the total dose of prednisolone they would administer (in mg) and its frequency; we calculated the dosage in our analysis. Although the range of selected dosages was similar to before, comparison of paired responses from the same individuals showed both BCCs (p = 0.002) and those in PCP (p = 0.025) chose significantly lower dosages for the 40 kg dog compared to the 9 kg dog (**Fig 5C**). However, this finding was complicated by the variability of individual responses in both groups (**Fig 5D**). In both scenarios, more BCCs and veterinarians in PCPs opted to administer prednisolone twice daily compared to once daily. This decision was largely consistent across both questionnaire scenarios, though 11/54 (20.4%) of those in PCPs who chose to administer prednisolone once daily to the 9 kg dog decided to prescribe it twice daily to the 40 kg dog, suggesting veterinarians may prefer fractionated doses if the total daily dose is larger (**Fig 6**).

**Fig 7:**
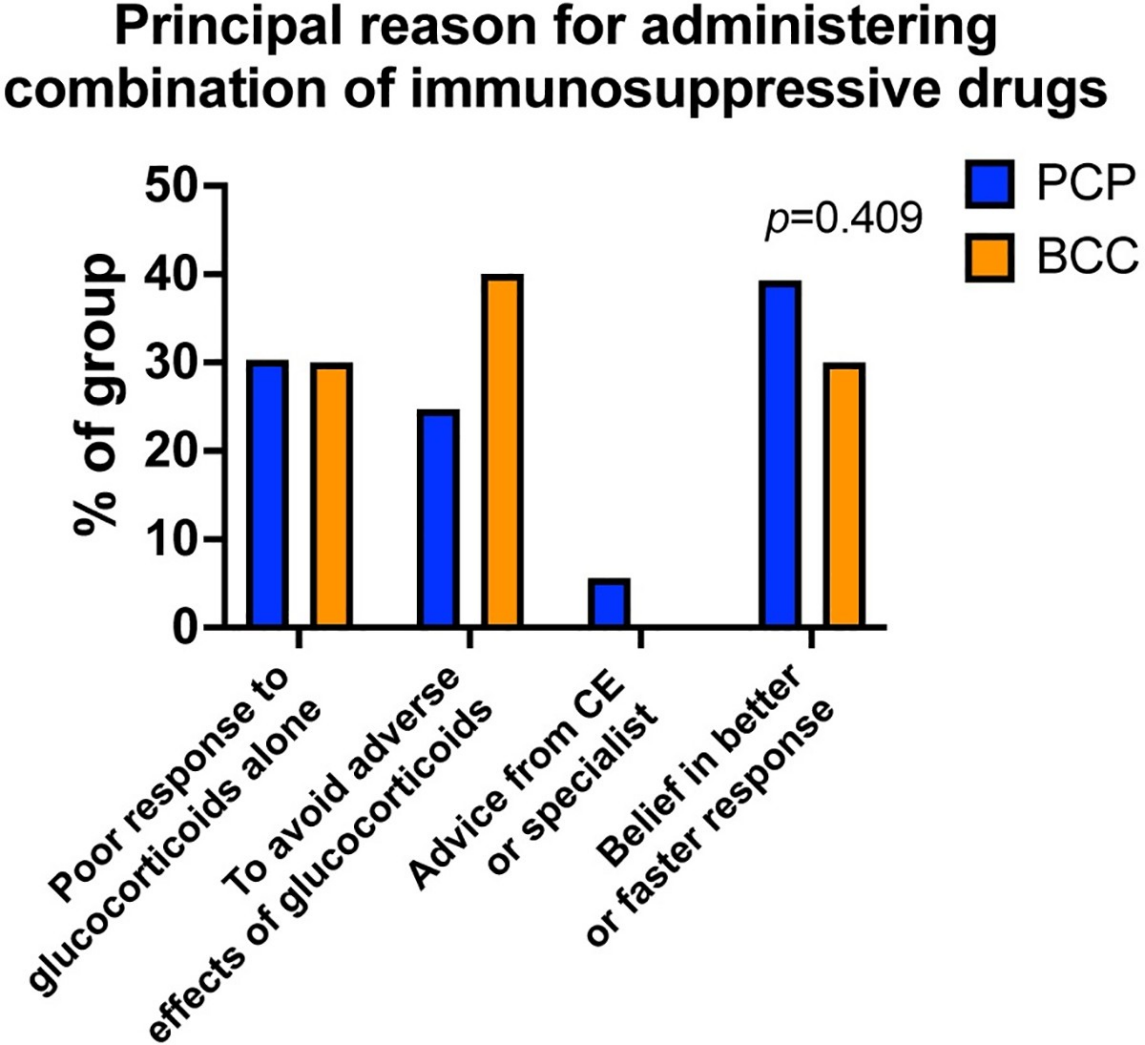
Primary care practitioners and board-certified clinicians use a combination of immunosuppressive drugs for different reasons in the clinic. Bar graph showing the percentage of respondents who administered a combination of drugs principally for the indicated reasons, separated according to group. PCP: primary care practitioner, n = 89. BCC: board-certified clinician, n = 20. CE: continuing education. Groups compared with Chi squared test.

Use of additional immunosuppressive drugs alongside glucocorticoids has become a controversial topic in the treatment of IMHA [6]. Of a total of 180 responses on this subject, 94 veterinarians in PCP (59.5%) stated they used a combination of immunosuppressive drugs, compared to all 22 BCCs (100%, p<0.0001). Principal reasons for using combination therapy were similar in PCPs and BCCs (p = 0.409), with the most frequent reason in PCP (35/89, 39.3%) being a belief this would achieve faster or more effective control of disease, whereas board-certified specialists most commonly used a combination to alleviate the adverse effects associated with glucocorticoids (8/20, 40.0%; **Fig 7**). In both groups, a similar proportion (55/91, 60.4% of those in PCP and 10/21, 47.6% of BCC, p = 0.283) indicated they treated with glucocorticoids initially and then introduced another drug later if the response was inadequate.

**Fig 8:**
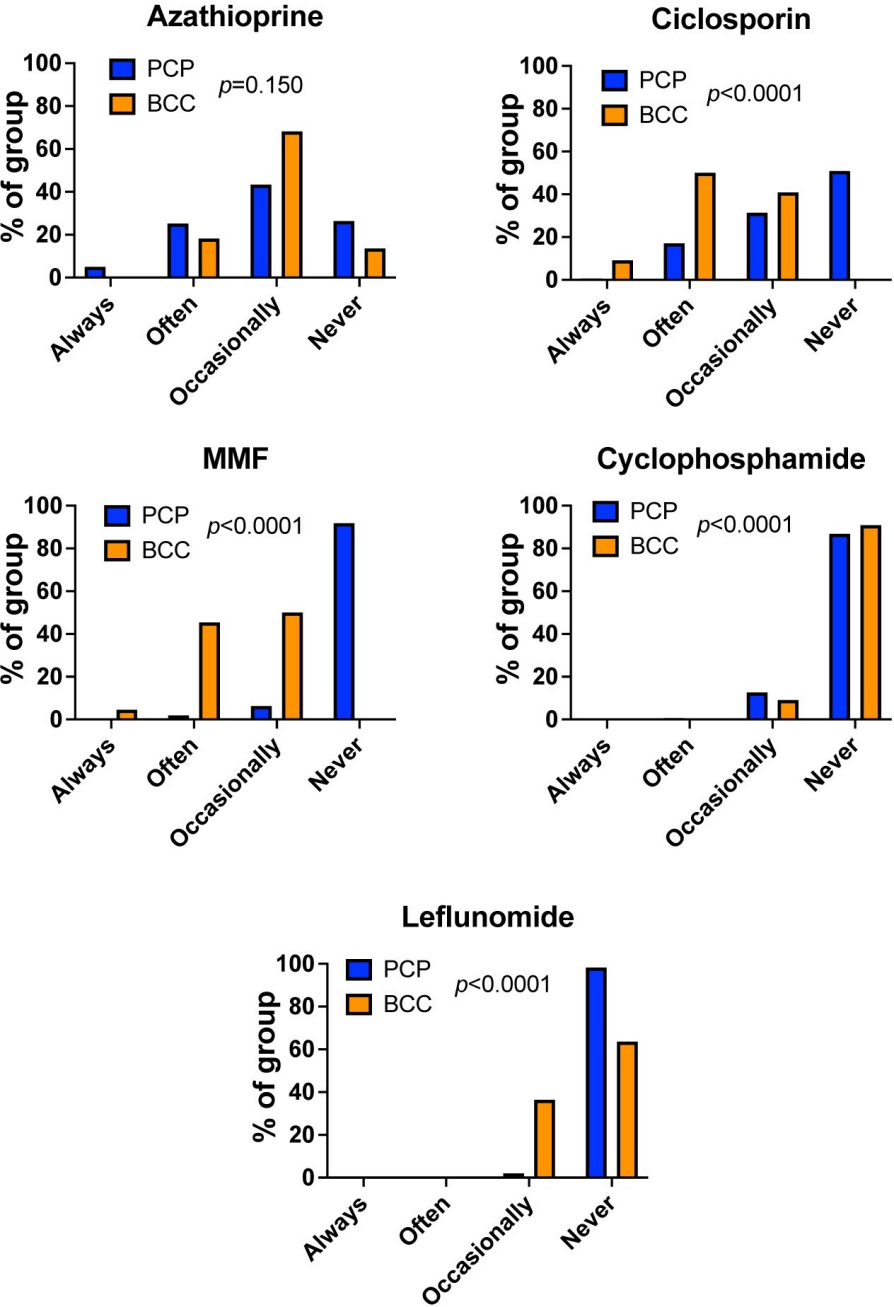
Primary care practitioners and board-certified clinicians use different combinations of immunosuppressive drugs in the clinic. Bar graphs showing the percentage of respondents using (**A**) azathioprine, (**B**) ciclosporin, (**C**) mycophenolate mofetil, MMF, (**D**) cyclophosphamide, or (**E**) leflunomide with indicated frequency according to group. PCP: primary care practitioner, n = 159. BCC: board-certified clinician, n = 22. Chi-squared or Fisher’s exact tests.

Of the immunosuppressive drugs used alongside glucocorticoids in dogs with IMHA, use of cyclophosphamide has been discouraged owing to a possible detrimental effect on outcome [7]. Whereas azathioprine has been available for decades in veterinary practice, other drugs, including ciclosporin [8], mycophenolate mofetil (MMF) [9], and leflunomide [10] have only been used in veterinary clinical practice more recently. This pattern appeared to be reflected in the responses to our questionnaire, with ciclosporin, MMF, and leflunomide all used more frequently by BCCs (all p<0.0001), whereas azathioprine was used at similar levels in both settings (p = 0.150; **Fig 8**). Cyclophosphamide was used often or occasionally by 21/159 (13.2%) of those in PCP and occasionally by 2/22 (9.1%) BCCs, with no significant difference between groups (p = 0.831).

**Fig 9:**
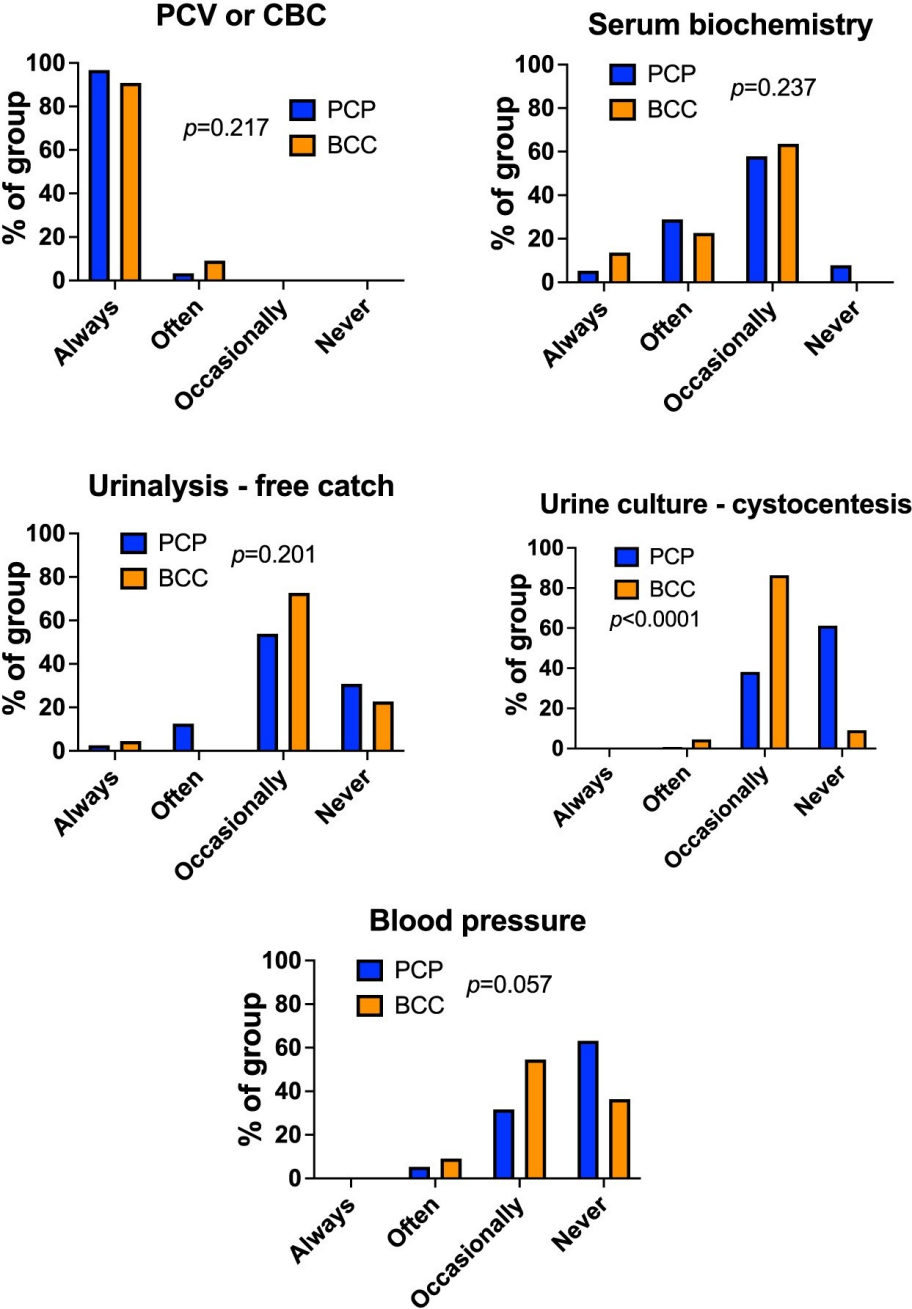
Board-certified clinicians obtain more urine samples by cystocentesis during follow-up visits. Bar graphs showing percentage of respondents performing indicated tests at indicated frequencies according to group during follow-up visits for dogs being treated for IMHA. PCP: primary care practitioner, n = 152. BCC: board-certified clinician, n = 22. Chi-squared or Fisher’s exact tests.

Respondents estimated that dogs with IMHA require immunosuppressive treatment for a median of 5.0 months (IQR: 3.5–6.0), with similar values provided by those in PCP (median 5.5 months, IQR: 3.5–6.5) and BCCs (median 5.0 months, IQR: 4.5–6). There was considerable variation in the narrative descriptions given by respondents on their approach to tapering drug doses over time and on the frequency of re-examination visits (S2 File). At follow-up visits, BCCs and those in PCPs undertook packed cell volume measurement or CBCs, serum biochemical profiles, urinalysis by free catch, and blood pressure measurements with similar frequency (p = 0.217, p = 0.237, p = 0.201, and p = 0.057 respectively), but BCCs were more likely to obtain urine samples for culture by cystocentesis (p<0.0001) (**Fig 9**).

### Antithrombotic treatment

**Fig 10:**
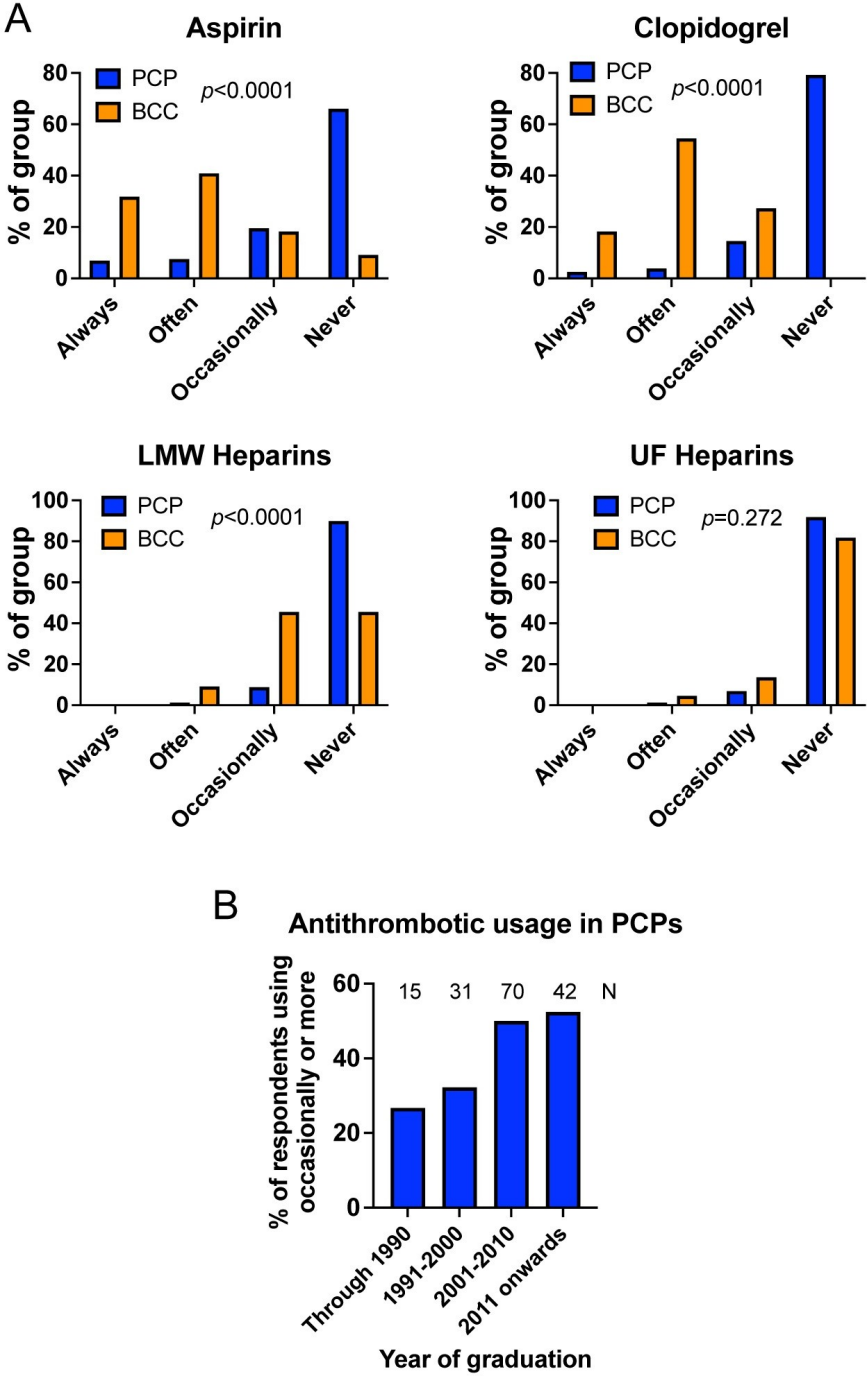
Antithrombotic treatment is not used by the majority of respondents in primary care practice. (**A**) Graphs show the proportion of respondents using aspirin, clopidogrel, low molecular weight (LMW) heparins, or unfractionated (UF) heparins. PCP: primary care practice, n = 159. BCC: board-certified clinician, n = 22. Groups compared with Chi squared or Fisher’s exact tests. (**B**) Proportion of respondents in PCP using any antithrombotic product at least ‘occasionally’ by year of graduation, annotated with number of respondents per group.

Immune-mediated haemolytic anaemia is associated with hypercoagulability [11] and an increased risk of thromboembolic disease [12], and thromboembolism is observed in a high proportion of dogs that die early in the course of disease [12]. Recent guidelines for treatment of IMHA recommend strongly that all dogs receive thromboprophylaxis alongside immunosuppressive treatment unless severely thrombocytopaenic [2,13]. However, among 181 individuals responding, only 93 (51.4%) indicated they ever used any antithrombotic drugs in dogs with IMHA. The frequency of this treatment differed according to setting, with 71/159 (44.7%) of those in PCP administering thromboprophylaxis at least occasionally, compared to 22/22 (100%) of BCCs (p<0.0001). This difference was apparent for prescribing of all forms of antithrombotic drug except for unfractionated heparins, which were rarely used by respondents from either group (**Fig 10A**). In addition to those drugs shown in **Fig 10A**, one BCC indicated that they occasionally used the oral factor Xa inhibitor rivaroxaban. Among those in PCP, the proportion of respondents administering antithrombotic medications increased with more recent graduation (**Fig 10B**).

### Vaccination in dogs with previous IMHA

In our practices, we are asked frequently by owners whether their dog with IMHA can be vaccinated, either while still receiving treatment or after maintaining complete remission for long periods of time. A previous study suggested recent vaccination could be a risk factor for the first occurrence of IMHA [14] and, though contradicted by a later study [15], we believe the notion that vaccination will cause relapse is widespread among veterinarians. To explore this idea, we presented a clinical scenario describing a dog in complete remission with no treatment for 6 months that is due to be vaccinated. Presented with this situation, 94 of 164 respondents (57.3%) opted to vaccinate, whereas 70 (42.7%) refused, reflecting a clear division in opinion. This decision was not significantly different between work settings (with 85/143, 59.4% of those in PCP and 9/21, 42.9% of BCC, p = 0.164, choosing to vaccinate). Some respondents (n = 20) indicated they would offer measurement of antibody titres, whereas others expressed a strong view that vaccinations are associated with relapse of IMHA (n = 18) or indicated they would treat the dog with glucocorticoids before and after vaccination to alleviate any risk of relapse (n = 2).

## Discussion

In this study, we show there are similarities and important differences in the clinical approach to treatment of IMHA in dogs in PCPs and referral hospitals. Whereas veterinarians in both settings performed diagnostic tests needed to confirm a diagnosis of IMHA with similar frequency, BCCs were much more likely to undertake extensive investigations for possible underlying causes of immune-mediated disease. Respondents in both groups reported use of glucocorticoids at similar dosages and with similar adjustments for bodyweight, and, although both groups used combination immunosuppressive treatment at similar rates when commencing therapy, BCCs reported always using a combination of drugs rather than glucocorticoids alone in cases they treated. Furthermore, reported use of antithrombotic treatment was limited in PCPs, particularly among practitioners that graduated earlier, but was used more extensively by BCCs.

Others have speculated that dogs with IMHA treated at referral hospitals represent a subset with more severe disease [16]. If true, this could confound our observations because BCCs and those in PCP could be investigating and treating different subtypes of IMHA, resulting in differences in clinical approach. While our study suffers from a lack of objective data to determine whether cases treated in these different settings were similar at diagnosis, we suspect there is considerable overlap between these groups because those in PCPs reported 90% of dogs were being treated exclusively in that setting, with more than 25% not referring any dogs with IMHA. If cases seen by BCCs were more severely affected, we would also expect intensity of treatment to be greater in that group, whereas, if anything, the opposite appeared to be true. For example, BCCs rarely used a starting dosage of prednisolone above 2 mg/kg per day and most often used another drug to limit glucocorticoid-related adverse effects. Additionally, we did not ask respondents about situations that would be unique to primary or specialist care, with all scenarios and use of all named tests and drugs being feasible in either setting. Therefore, we suggest that differences observed in this questionnaire may represent genuine differences between settings in the approach to investigation and management of IMHA in dogs, but further objective data are needed to determine whether disease severity differs in referred dogs.

Authors of a recent ACVIM consensus statement recommended that various tests, including thoracic and abdominal imaging, be undertaken to detect concurrent diseases that might promote dysregulated immune responses to produce so-called ‘associative’ IMHA [3]. However, two recent studies indicate that dogs with IMHA fulfilling multiple diagnostic criteria rarely have abnormalities on thoracic imaging and often do not have findings of clinical significance on abdominal imaging, suggesting these tests have low diagnostic yields and may be dispensable in many cases [17,18]. Accordingly, we were interested to note that imaging was performed less commonly by those in PCPs in our study compared to BCCs, which probably reflects a difference in the perceived cost-benefit balance between these settings. We speculate that this difference could be attributable to differences in the severity or complexity of cases referred to specialist centres, to lower concern among those in PCPs for possible underlying causes of IMHA, or to a culture among BCCs that may set a higher value on ‘completeness’ of investigations.

Of the vectorborne pathogens suggested to have some association with IMHA in dogs, only *Anaplasma phagocytophilum* is endemic in the UK, and the level of evidence linking this pathogen with disease is considered to be low [3]. However, autochthonous cases of *Babesia canis* [19] and *Ehrlichia canis* [20] have been reported recently in untravelled dogs, suggesting new pathogens may become established with changes in climate that permit survival of new tick species and with increases in international movement of dogs. We were therefore interested to note that BCCs more often performed testing for vectorborne agents in dogs that did not have a history of travel outside the UK, suggesting BCCs may be more aware of or concerned about emerging infectious diseases than those in PCPs.

Individuals pursuing specialist training participate in standardised programmes that require candidates to be familiar with recent scientific literature. We believe this may explain the remarkable consistency in responses from BCCs, with most appearing to undertake similar diagnostic investigations, treat cases similarly after diagnosis, and make similar decisions in clinical scenarios. In PCPs, exposure to these materials may be more limited, possibly explaining the greater variability in some parameters, such as the starting dosage of prednisolone. These factors could also explain the difference in use of antithrombotic treatment between settings, even though, in our experience, drugs such as aspirin or clopidogrel are stocked by most PCPs. We were interested to note the increasing use of antithrombotics in those graduating after 2000 because the first major study to describe use of aspirin for dogs with IMHA was published in 2005 [21], meaning those graduating earlier may not be familiar with its contents from their university education.

We found approaches to pre-transfusion testing varied considerably, with some clinicians choosing not to perform blood typing of donor or recipient before transfusion. This could be attributable to limited availability of blood typing kits in some practices or could be a conscious choice because transfusion reactions have not been described for transfusion naïve dogs receiving their first DEA1 mismatched transfusion. However, because dogs with IMHA may require multiple transfusions, others have recommended that blood typing before transfusion can be used to prevent sensitisation of DEA1 negative recipients to DEA1 positive blood, avoiding the risk of severe haemolytic reactions if a sensitised dogs receives a mismatched transfusion [22]. Similarly, some clinicians indicated they would not perform crossmatching in dogs requiring a subsequent transfusion, which is likely to increase the risk of haemolytic reactions [23].

Glucocorticoids are used widely in veterinary practice, with approximately 25% of non-vaccine consultations in PCPs in the UK resulting in their systemic administration [24]. However, use of glucocorticoids at immunosuppressive dosages produces severe adverse effects, which may impair quality of life [8,25–27]. For this reason, in the recent ACVIM consensus statement on treatment of IMHA, panel members recommended against exceeding an initial dosage of 3 mg/kg per day, with some indicating they did not see an indication for more than 2 mg/kg per day owing to the presumed risk of severe adverse effects without any additional clinical benefit [2]. Despite this, some in PCPs reported using dosages as high as 5–8 mg/kg per day, with practitioners graduating earlier also using greater dosages, whereas those graduating after 2000 were more likely to use dosages of 1.5 mg/kg per day or less. We suggest this may reflect a greater concern among those in PCP to control haemolysis, whereas BCCs might be more concerned by adverse effects, also leading them to use an additional immunosuppressive drug more frequently for this purpose. By highlighting these differences, we do not wish to pass judgement on veterinarians working in PCPs because there is no published evidence comparing outcome for dogs with IMHA treated with different dosages of prednisolone. Instead, we wish to emphasise that individuals in different settings are apparently reaching different conclusions in their cost-benefit analysis on these questions, which probably reflects differences in the perceived importance and awareness of different aspects of the disease.

Many BCCs and practitioners in PCPs made allowance for bodyweight when selecting doses of prednisolone for initial treatment of dogs with IMHA. This is important because metabolic rate is more closely related to surface area than bodyweight [28], meaning that lower total doses are required to achieve the same blood concentrations of prednisolone in larger dogs [29]. Consequently, dosing according to surface area may be an effective practice to limit adverse effects in larger dogs, as recommended previously [2].

In corresponding with owners and veterinarians, a topic we encounter frequently concerns the vaccination of dogs that have recovered completely from IMHA, and our study confirmed a striking difference of opinion regardless of practice setting. Aside from published evidence, this debate is also affected by other information, including global anti-vaccine sentiments and information provided by vaccine manufacturers. Many respondents to our questionnaire indicated they would offer measurement of antibody titres against pathogens composing the major vaccines, but this approach is not suitable for some pathogens with labile serological responses, such as *Leptospira spp*. Uncertainty on this topic warrants further, prospective investigation of the risk of relapse after vaccination in dogs with IMHA, weighed against the occurrence of infectious diseases in those not vaccinated. A recent survey-based retrospective study suggested there was no increased risk of reactions in dogs with IMHA receiving vaccines after diagnosis, but this study may have been underpowered because relapse after finishing treatment occurs in only a small proportion of dogs with IMHA [30].

In a cross-sectional study, it is not possible to determine whether associations are causal because they could be confounded by unmeasured variables. Therefore, while we might speculate that differences between those in PCP and BCCs could be attributable to specialist training, this cannot be demonstrated without longitudinal data to compare treatment approaches of the same individuals before and after training. Our study has a number of additional limitations, including a lack of qualitative interviews on the controversial topics identified and a lack of objective data. Respondents to the questionnaire could have been veterinarians with a particular interest in this subject or particular enthusiasm for participating in surveys, both of which could introduce bias in our results. We offered only the option of a computer-based questionnaire for respondents, when other individuals might have responded if they could have completed a paper copy. We were concerned that respondents might answer questions based on theoretical knowledge rather than clinical acumen, leading us to include clinical scenarios. However, these provided limited descriptions of clinical situations, with no facility to request further information, as would be possible in reality. Consequently, responses could have been biased by our descriptions or the wording of the scenarios and may not reflect accurately the decisions being made by clinicians. Our scenarios and other parts of the questionnaire neglected the opinions of owners on the treatment of their dogs, which is an essential component of clinical decision-making that would affect many of the situations we described. Although we compare responses to our questionnaire with information provided in the ACVIM consensus statements, the publication of these statements could have changed clinical practice since the questionnaire was completed, highlighting the importance of repeating our survey in future to determine whether guidelines are disseminated effectively. Finally, while statistical tests used in this study are not affected by unequal group sizes, the difference in the relative number of veterinarians in PCP and BCCs may have increased the risk of type II errors. This means further differences between those in PCPs and BCCs could have been apparent if the group sizes were more equal, and the small number of responses from BCCs in this study is a clear limitation. Additionally, the lack of responses from those clinicians specialising in emergency and critical care deprives this study of an important contribution from a group of clinicians who also manage dogs with IMHA.

Taken together, the results of our study reveal differences in the treatment of dogs with IMHA in PCPs and referral practices, which may have important consequences for the design and applicability of future research. We show treatment decisions may differ with time since graduation in PCPs, suggesting there may be a trade-off between clinical experience and efforts to implement recent scientific literature. Finally, our results reveal a striking lack of consistency in treatment intentions for IMHA, highlighting a clear need for effective dissemination of published clinical evidence through provision of clinical guidelines, checklists, implementation of the clinical audit cycle, and continuing education.

## Supporting information

S1 File Complete transcript of the questionnaire used in this cross-sectional study. (DOCX)

S2 File Table of all questionnaire responses. Note that all information that might be used to identify respondents has been removed for data protection purposes, including post code, IP address, gender, specific list of post-nominal letters, and country of residence and graduation.
(CSV)
