# Supplementary material for: Cross-sectional study of approaches to diagnosis and management of dogs with immune-mediated haemolytic anaemia in primary care and referral veterinary practices in the United Kingdom
Source: PLoS One. 2021 Sep 20;16(9):e0257700. doi: 10.1371/journal.pone.0257700 (PMC8452064; doi:10.1371/journal.pone.0257700)
Supplement: S1 File — (DOCX) [file pone.0257700.s001.docx]

**S1 Supplementary Information**: IMHA Treatment Questionnaire – Text of questions

1. Where in the UK is your practice located? Please enter the 3 first letter of your post code.
   1. Free text response
2. How many full-time equivalent (FTE) veterinary surgeons are employed by the practice (e.g. one full time vet = 1.0 FTE, one part-time vet = 0.5 FTE)
   1. Free text response
3. What is your gender?
   1. Responses: Male, Female, Prefer not to say
4. In which country did you obtain your veterinary degree? Please enter country.
   1. Free text response
5. What was your year of graduation? Please enter year.
   1. Free text response
6. Do you have a post-graduate veterinary clinical qualification? Please enter your post-graduate qualification.
   1. Free text response
7. How many dogs have you diagnosed with IMHA in the past year?
   1. Responses: None, 1-2 cases, 3-5 cases, 6-10 cases, >10 cases
8. What percentage of these cases have started to receive treatment after the initial diagnosis?
   1. Free text response
9. How long ago did you diagnose your most recent case of IMHA?
   1. Responses: Less than 2 weeks ago, Less than 1 month ago, Less than 3 months ago, Less than 6 months ago, 6-12 months ago, >12 months ago
10. Which tests do you perform to reach a diagnosis of IMHA?
    1. Responses: Always (100%), Often (>50%), Occasionally (<50%), Never (0)
    2. Tests: Haematology at an external laboratory, Haematology in house, In house blood smear evaluation, In-saline agglutination test, Coombs’ test, Serum biochemistry, Urinalysis, Other (please specify)
11. Which diagnostic tests do you perform to rule out underlying causes of IMHA?
    1. Responses: Always (100%), Often (>50%), Occasionally (<50%), Never (0)
    2. Tests: Serum biochemistry, Urine culture, Faecal analysis, Abdominal imaging, Thoracic imaging, Other (please specify)
12. Do you test for infectious diseases (including Borrelia, Ehrlichia, Anaplasma, Babesia and Leishmania)?
    1. Responses: Always regardless of travel history, Always if there is a travel history, Often if there is a travel history, Occasionally if there is a travel history, Never regardless of travel history, Other (please specify)
13. If you work at a first opinion practice, what percentage of dogs with IMHA do you refer for specialist treatment?
    1. Free text response
14. What are your main reasons for referral? (Please enter all that apply)
    1. Free text response
15. Do you administer blood transfusions in your practice/institution?
    1. Responses: Yes, No
16. Do you perform blood typing prior to transfusion?
    1. Responses: Both recipient and donor are typed, Neither recipient nor donor are typed, The donor is typed, The recipient is typed
17. Do you perform cross matching prior to blood transfusion?
    1. Responses: Before every transfusion, If the dog has received a previous transfusion, Never
18. What is the source of blood products for your practice/institution?
    1. Responses: Pet Blood Bank, Donor animals owned by practice or staff, Donor animals owned by clients,
19. Which of the following drugs do you use to treat dogs with IMHA?  Please indicate the dose used.
    1. Responses: Always (100%), Often (>50%), Occasionally (<50%), Never (0)
    2. Drugs: Corticosteroid (e.g. prednisolone, dexamethasone), Cyclosporine, Azathioprine, Mycophenolate mofetil, Leflunomide, Cyclophosphamide, Human gamma globulin, Aspirin, Clopidogrel, Heparin (unfractionated), Heparin (low molecular weight), Other (please specify)
    3. Option to add dosage in free text
20. If you could use only one of the drugs listed above for treatment of a dog with IMHA, which would you choose?
    1. Free text response
21. Do you use a combination of immunosuppressive drugs for treatment of dogs with IMHA?
    1. Responses: Yes, No
22. Do you administer any other types of drug to dogs with IMHA?
    1. Free text response
23. What combination of immunosuppressive drugs do you use for treatment of dogs with IMHA?
    1. Free text response
24. What is the main reason for using a combination of immunosuppressive drugs?
    1. Free text response
25. When do you start the 2nd immunosuppressive drug?
    1. Responses: At the time of treatment start, Later
26. Please describe how you would change the doses of each immunosuppressive drug over time?
    1. Free text response
27. Please describe how frequently you re-examine dogs with IMHA after diagnosis?
    1. Response: Every [enter number] [enter time interval, e.g. days, weeks, months]
28. Which of the following tests do you perform at follow-up visits?
    1. Responses: Always (100%), Often (>50%), Occasionally (<50%), Never (0)
    2. Tests: Packed cell volume or haematology, Serum biochemistry, Urinalysis (free catch), Urine culture (cystocentesis), Blood pressure measurement, Other (please specify)
29. Please estimate the length of time for which dogs with IMHA require immunosuppressive treatment.
    1. Free text response
30. If euthanasia is requested by the owner of a dog with IMHA, please indicate the most common reason for this.
    1. Free text response
31. When discussing a dog with newly diagnosed IMHA with their owner, which words would you use to describe the prognosis?
    1. Free text response

**Clinical Scenarios (shown one at a time)**

1. A 5 year old, male neutered Labrador was diagnosed with primary IMHA 8 days ago.  On initial presentation, the PCV was 12% and the dog received a transfusion of packed red blood cells, which raised the PCV to 18%.  He subsequently began to receive prednisolone at 3 mg/kg once daily. He has received the same dose of prednisolone for the last 7 days and his PCV today is 28%. How would you advise the owner regarding further treatment?
   1. Responses: Reduce the current dose, Continue with the current dose, Increase the current dose
   2. Instruction to state the new dose in mg and frequency
2. You are presented with a 4 year old Welsh Springer Spaniel who was diagnosed with IMHA last year.  He was treated successfully and stopped receiving treatment for this disease 6 months ago.  He is due for vaccination today. How would you advise the owner?
   1. Responses: Do not vaccinate the dog today, Vaccinate the dog today.
   2. Instruction to provide an explanation of the decision in free text.
3. You are seeing a 4 year old English Springer Spaniel for re-examination.  The dog was diagnosed with primary IMHA several weeks ago.  The dog has been receiving prednisolone at 1.5 mg/kg once daily for the last 3 weeks and the current PCV is 42%.  The bodyweight is 17 kg.  You have decided to reduce the dose of prednisolone at this visit. Please indicate the total amount and frequency of administration of prednisolone (i.e. xx mg xx times per day) that you would prescribe for this dog.
   1. Free text response
4. You have just diagnosed a 7 year old, female neutered Rottweiler with primary IMHA.  Her current weight is 40 kg.  You wish to start treating this dog with prednisolone. Please indicate the total amount and frequency of administration of prednisolone (i.e. xx mg xx times per day) that you would prescribe for this dog.
   1. Free text response
5. You are presented with a 5 year old Border Collie who was diagnosed with primary IMHA several months ago.  Treatment with prednisolone was stopped 8 weeks ago, but the dog has continued to receive azathioprine (at 25 mg per metre squared every other day).  The PCV today is 48%. How would you advise the owner regarding further azathioprine treatment?
   1. Responses: Stop treatment now, Taper the dose slowly over several weeks.
6. You are examining a 6 year old German Pointer who was diagnosed with primary IMHA several weeks ago.  Three weeks ago, the PCV was 42%, and the dose of prednisolone was reduced to 1 mg/kg once daily.  The PCV today is 30% and the dog is clinically normal.  The bodyweight is 22 kg.  What would you advise regarding further treatment of this dog?
   1. Responses: Increase the dose of prednisolone, Continue with the same dose of prednisolone (1 mg/kg per day) – please specify the duration below
   2. Instruction to provide new dose in free text.
7. You are examining a 6 year old Cocker spaniel who was diagnosed with primary IMHA several weeks ago.  The dog has been receiving prednisolone at 1 mg/kg once daily for the last 3 weeks.  The current PCV is 42% and you have decided to reduce the dose of prednisolone.  The bodyweight is 15 kg. Please indicate the total amount and frequency of administration of prednisolone (i.e. xx mg xx times per day) that you would prescribe for this dog.
   1. Free text response.
8. You have just diagnosed a 3 year old, male entire Dachshund with primary IMHA.  His bodyweight is 9 kg.  You have decided to start treating the dog with prednisolone. Please indicate the total amount and frequency of administration of prednisolone (i.e. xx mg xx times per day) that you would prescribe for this dog
   1. Free text response
