# Supplementary material for: Cross-sectional study of approaches to diagnosis and management of dogs with immune-mediated haemolytic anaemia in primary care and referral veterinary practices in the United Kingdom
Source: PLoS One. 2021 Sep 20;16(9):e0257700. doi: 10.1371/journal.pone.0257700 (PMC8452064; doi:10.1371/journal.pone.0257700)
Supplement: Attachment — Submitted filename: Response to Reviewers.docx [file pone.0257700.s003.docx]

We are grateful to all the reviewers for their comprehensive consideration of the manuscript and constructive comments for its improvement. Please note that line numbers in this response to reviewers refer to the version of the document with tracked changes.

We wish to declare an error in the previous version of the manuscript, in which we stated that 25 BCCs in internal medicine completed the questionnaire with no responses from ECC specialists. On reviewing the raw data, we find there was 1 ECC boarded specialist, who was incorrectly coded as an internal medicine specialist in our analysis. This does not change the results of comparisons between PCP and BCCs because this individual was always included in the BCC group, and we have altered the manuscript where appropriate to make it clear we had a single ECC response.

Reviewer #1: PONE-D-21-17816

This manuscript describes the results of a survey about the diagnosis and treatment approach to IMHA completed by primary care practitioners and board certified internal medicine specialists in the UK. The study has some interesting findings, but I believe that more information is required before the manuscript is ready for publication.

Firstly, it is a bold choice of the authors not to do any statistical analyses of their data. I do understand the rationale, but it makes hypothesis testing difficult. I can accept the lack of statistical analyses as long as all of the raw data (or at least the proportions) are available such that a reader could choose to do their own analyses if they wished. Although the authors stated that their raw data is available in the manuscript or in the supplemental data, I cannot see any files containing the raw data (in xls or csv format) attached to the submission. Additionally, the authors do not specifically refer back to their hypotheses in the discussion section so it is unclear how they used their descriptive data to address their hypotheses.

Thank you for these comments. We have now provided all raw data as supplementary file S2. Based on other reviewer comments, we have introduced statistical comparisons for some of the main findings in the study.

Title
• The title should be adjusted to clarify that this study focused on veterinarians in the United Kingdom only

Changed as suggested.

Introduction:
• Line 62 – can you clarify of what you mean by “misaligned with principles” – I would argue that all veterinarians operate on the same principles of providing accessible and high quality veterinary care to pets and their owners

We agree with the reviewer that broad principles of treatment in veterinary medicine are shared. In this sentence, we meant that consensus guidelines sometimes recommend forms of treatment and investigation that are more intensive than might always be appropriate/desired by clients in primary care practice. We have re-phrased to make this clearer (line 65).

• Line 67 – I do think it is worthy of mention though that veterinarians are still trained at a limited number of veterinary schools, and generally trained by BCCs. If not mentioned in the intro, please address this in the discussion (paragraph lines 368-379)

We have added this in line 69.

• Similarly, it appears that you collected data about which country veterinarians graduated from – it may be interesting to include how many of the surveyed veterinarians graduated from UK schools vs from other countries

We have added this information under ‘Demographic Characteristics’ in the Results section, line 172.

Materials and Methods:
• Line 85 – Can you please clarify that this was to “veterinarians in PCPs and BCCs in the UK…”

Changed as suggested, line 96.

• Can you please clarify somewhere in the M&M section whether or not the participants were able to navigate backwards and forwards in the survey (and change their answers) – as this may have led to the case scenarios having the potential to influence the respondents answers more than if they could only move forward in the survey

Participants could go back – this is added in line 111.

• Line 104 – again presumably this is a mailing list maintained by a university in the UK?

This is correct – it was the RVC database, now added on line 119. As a side note, we very strongly believe in blinded peer review, which is why we formatted the manuscript appropriately for this, even though this journal does not support this policy.

• Line 106 – and a national database in the UK?

Yes – we have added the specific information, line 120.

• Line 112 – can you please reference these regulations

Added in line 127.

• Line 117 – can you please provide more information about how vets are included in www.specialists.com – it appears that perhaps this includes all of the ACVIM listings. What about capturing DACVECCs and DECVECCs? I would imagine that there are well over 20 ECC specialists in the UK

Any American boarded specialist is included in [www.specialists.com](http://www.specialists.com) for all colleges (ACVIM, ACVECC, ACVS etc) if they consent to be listed, so this allowed us to capture ACVECC diplomates in the UK. ECVECC was only founded in 2014 and initially was only composed of 10 invited diplomates in Europe; of these, all the UK-based diplomates were also ACVECC boarded. We have checked the current diplomate list for ECVECC (July 2021), and all UK-based members are also ACVECC boarded, so we don’t think anyone was missed. Actually, we believe it is accurate that there were only 8 ACVECC diplomates in the UK at the time of the survey – the ECC specialist structure has historically been much less developed than in the US compared to internal medicine. There are certainly not 20 ECC diplomates in the UK now.

• Line 127 – countries is probably a better word here than “territories”

Changed as suggested, line 143.

• Line 155 – here you state the number of cases the survey respondents “treated in the previous year” whereas it appears that your survey (Q7) only askes how many dogs they diagnosed with IMHA in the last year – these are potentially different numbers (either because a colleague did the diagnosis but the participant was involved in treatment, or a case was diagnosed but then euthanised without treatment) – please clarify

The reviewer’s point is correct, and we have changed the text accordingly, line 193.

• In Figure 2 you use the term “direct antiglobulin test” but it appears you say “Coombs’ test” in the survey – I would recommend keeping this consistent with the survey terminology

We agree and have changed to Coombs’ test throughout.

• Line 201 – “pre-transfusion testing” would be more appropriate than “procedures for administration”; the latter I would consider to include administration via a pump or no pump, rate, monitoring etc.

Changed as suggested, line 275.

• Line 239 – please clarify here that you mean lower dosages on a mg/kg basis (rather than lower total doses)

We believe this is clear in the text and figure 5C – in both we refer only to dosage not dose.

• Lines 248-255 – this section about once vs. twice daily dosing is likely affected by the fact that all of the scenarios in your survey reported once daily doses – please address this as a limitation in your discussion section as this information may have biased how the respondents answered

We have added this as a limitation, but we feel the likely influence of this is very small, particularly since a high proportion of respondents said they would dose twice daily. If the scenarios were causing some bias, we might expect there would be a higher frequency of once daily dosing than seen here. Additionally, respondents only saw one scenario at a time in the survey unless they specifically chose to go back, and we suspect they would not recall such specific details from scenario to scenario.

• Line 293 – I think it needs to be addressed here that not only is TE a risk but that it is believed to be the most common cause of death in dogs with IMHA

We have expanded this sentence, line 410.

• Line 295 – please also reference the CURATIVE guidelines

We have added as suggested, line 412.

Discussion:
• Please refer back to your hypotheses in your discussion section – with consideration of how you can “test” those hypotheses without any statistical comparisons

We now add statistical comparisons to provide support for this.

• The lack of ECC specialists in the survey needs to be addressed as a potential limitation, since you had intended to include this group – based on the respondents it is really a survey about BC internal medicine specialists (rather than all BCCs that routinely treat IMHA)

We have added this as a limitation (line 601).

• Please include as a limitation that your survey did not ask about whether or not respondents were using direct oral anticoagulants (eg. rivaroxaban) for treatment of IMHA

Respondents had the option to add the details of any other antithrombotic drugs used in the free text ‘other’ box included in that question. Only 1 BCC indicated that they used rivaroxaban occasionally. We have added this information in the text of the results (line 420) but do not think this needs to be included as a limitation because we gave respondents the opportunity to provide this information.

• Line 405 – reference laboratories do report what they consider to be protective titers – so please expand on this a little

We agree this is provided for many of the pathogens for which vaccines are commonly administered. We specify that this approach would not be suitable for leptospirosis, line 558.

• You mention the ACVIM consensus statement in your paper, but I do think that there are more opportunities to refer back to this statement and provide clarity to the reader.

For the following points about the discussion, we agree with many of the comments raised by the reviewer but, in some cases, we have made few changes to the manuscript because 1) this study is a cross-sectional survey about recent approaches to treatment of IMHA, which does not provide any new data about the most appropriate way to treat dogs with IMHA. Therefore, it does not seem appropriate to us to write long sections in the discussion about how IMHA is most effectively treated when the results section does not contain any data on this subject; 2) as the reviewer points out, there are many resources providing recommendations for different aspects of the care of dogs with IMHA, and we do not feel it is necessary to replicate this information in our manuscript. Instead, we try to reference these resources more effectively; 3) some aspects of the treatment of IMHA are widely accepted in the community of board-certified specialists but are not actually supported by any evidence. Therefore, we feel it is difficult to state that particular practices are ‘correct’ or ‘incorrect’ without being able to provide any stronger evidence than the ACVIM consensus statements, which are acknowledged by the panel members to be based in many areas on clinical experience alone; 4) we have tried to make sure that the manuscript is not explicitly judgemental or critical of those working in PCPs because we feel it would be counterproductive, and some of the comments made by the reviewer would effectively force us to say that those in PCP are not doing a good job in treating dogs with IMHA; and 5) attempting to cover every topic, especially when there is no point of contention, makes the discussion unfocused and excessively long.

For example
o How do the diagnostic tests performed to identify IM erythrocyte destruction and hemolysis by the respondents allow them to obtain a “diagnosis” of IMHA Vs. being “supportive” of a diagnosis of IMHA or just “suspicious” of a diagnosis of IMHA

This system did not exist at the time the questionnaire was distributed, but we reviewed the test selection of individual respondents and classified them according to whether they always did sufficient tests to be able to place cases in the ‘diagnostic’ or ‘supportive/suspicious’ categories. The supportive/suspicious categories could not be separated because this depends on whether the test results actually show evidence of haemolysis or not. See line 212 onwards and new figure 2B.

o If survey respondents aren’t doing in-house or external haematology how are they even diagnosing anaemia? Presumably PCV/TS? This needs to be addressed

We agree this is an important question, but it is not something we can address with our data. We did not ask what tests respondents might perform as an alternative to complete CBC, so we cannot state how else they might be diagnosing anaemia. However, we suspect the apparent discrepancy is attributable to the format of the question in the survey, where we asked in the same grid whether respondents performed internal or external CBC. Therefore, a respondent might say they completed internal CBC often and external CBC often, but actually mean that they always complete a full CBC in every dog with IMHA, just at different labs. When we looked at the tabulation, 202/217 respondents in this question stated they always performed one or both types of CBC, and this is reflected in Fig 2A where the percentages always performing each type of CBC are complementary.

o How the reasons for your respondents giving a 2nd immunosuppressive drug compare to those advocated by the ACVIM consensus statement

Again, this is an interesting question but not something we can answer with our data. First, to clarify, the ACVIM statement does not advocate use of 2^nd^ drugs in any scenario. Instead, the statement indicates that there is insufficient evidence to determine whether use of a 2^nd^ drug will produce a more favourable outcome than glucocorticoids alone, and that either course of action is therefore appropriate. Based on the clinical experience of the panel, some scenarios are presented in which use of a 2^nd^ drug might be considered more seriously, but these scenarios have specific criteria that we cannot evaluate in our survey data. For example, many respondents stated that they used an additional immunosuppressive drug if the response to glucocorticoids was inadequate, but we do not have enough resolution in our responses to know if e.g. the respondent would wait for 7 days to see if the initial drug was effective or if the dog met the suggested criteria for PCV changes that would trigger a second drug, as outlined in the statement.

o Why body weight of the patient matters with regard to mg/kg pred dosage – this needs to be addressed more explicitly in case the reader isn’t familiar with why lower mg/kg doses are appropriate for large breed dogs

We have added some referenced remarks about the pharmacokinetics of prednisolone in dogs (line 545), but we are cautious about giving any recommendation ourselves because there has never been a comparison of the efficacy of treatment and adverse effect profile of these approaches.

o Did you give the respondents the opportunity to state whether or not they have used aspirin in combination with clopidogrel or a heparin in combination with aspirin/clopidogrel

We do have these data – the question was in the format of a grid where respondents could indicate all the drugs they used, including any we did not list by name. We reviewed the tabulation, but this does not show any consistent patterns of combined usage, so we have not added these data. The complete data are also available in S2 supplementary information for each participant to see which combinations were reported.

o The fact that the consensus statement was published after your survey, and the potential for the publication of such a statement to affect practice

We add this as a limitation of the study, also addressing the limitation noted below about dissemination of information, line 596.

• Can you comment on how the use of vector-borne disease testing by survey respondents relates to a. the prevalence of vector-borne diseases in the UK and ACVIM consensus recommendations for this testing

We have expanded the section on tests for underlying disease to include some discussion on this topic (see lines 232 and 493). Of the common vectorborne infectious agents, only *Anaplasma phagocytophilum* is endemic in the UK but uncommon, with a small outbreak of *Babesia canis* in 2016 in untravelled dogs in one very localised area that has not recurred. There have been 2-3 cases of *Ehrlichia canis* in untravelled dogs in recent years. Therefore, we feel the most common scenario (for respondents to often or always do testing in dogs that had a history of travel to an area where other agents are endemic) is completely in line with the ACVIM recommendations to test according to local conditions, and we speculate that BCCs might be more aware of emerging threats, which might explain why they test more often in untravelled dogs.

• With regard to use of pre-transfusion blood-typing and crossmatching please consider adding a section into the discussion about the potential consequences (based on the literature) of giving type mismatched blood, or not performing crossmatching for second and subsequent transfusions. Consider also referring to the recently published AVHTM TRACS guidelines

We have added a section to this effect, line 515.

• More discussion is also required about the potential adverse effects of very high doses of prednisolone so that the reader doesn’t go away thinking that its reasonable to give 5-8mg.kg of prednisolone. While I agree that we don’t know the optimal dosing strategy for dogs with IMHA, we do know a bit about what doses are needed to optimise immunosuppression

We feel this message was clear where we said that most clinicians would not exceed a dosage of 2 mg/kg per day, but we have expanded this sentence to state clearly that this is due to the presumed risk of adverse effects with no additional clinical benefit. As the reviewer states, there is no published evidence to support this assumption. See line 532

• Line s 435-436 – since your survey was prior to consensus guidelines for the diagnosis and treatment of IMHA, I don’t think that you can comment back the lack of effectiveness or dissemination of the guidelines. This would probably require before and after assessment to see if practice has changed

We agree and we have changed the wording to ‘published clinical evidence’, which encompasses e.g. research on antithrombotic drugs. See line 612.

• I would love to see the authors be a bit more clear in their future directions / how they intend to apply this information

We conclude the manuscript by indicating that clinical governance interventions are needed on this topic. See line 612.

Reviewer #2: This manuscript is well written and provides important information about the treatment habits in primary and specialty practice of an important veterinary disease: immune-mediated hemolytic anemia in dogs. I thought the authors did a good job of clarifying in the introduction why such information is useful, and were tactful in their discussion of reasons for differences between PCPs and BCCs. In general, conclusions are supported by the data presented, but the manuscript is significantly limited by the lack of statistical comparison between groups. The reviewer appreciates the differences in group sizes, but some statistical basis for statements like those found in lines 175-178: "However, BCCs were much more likely to undertake additional tests to establish if the IMHA was associated with an underlying cause..." would support the sentiments presented. Based on the values shown, the reviewer suspects many of these differences would be significant, and this would substantiate the discussion. The other primary concern is the low number of specialist responses reported. Were there really only 8 ECC specialists able to be contacted? The number of internists also seems low, and the low response rate for specialist veterinarians should be acknowledged as a significant limitation. It would be ideal to gather more specialist responses if possible. The reviewer was also interested to note that several questions in the survey were aimed at assessing drug tapering practices, but none of this data was presented in the manuscript, even in generalizing statements. This data would be of interest. The authors do not seem to have a supplementary file with all survey responses as seems preferred by the journal, or a mention of data being available upon request.

Thank you for these comments. We have added statistical tests for important comparisons.

Please see also our explanation for reviewer 1 but yes, we do believe there were only 8 boarded ECC specialists in the UK at the time of the survey, and we believe the number of internal medicine specialists we contacted represented the majority of the total number in clinical practice, of whom we believe there are 90 in the UK right now, with some expansion of this sector in the last few years. We have added the small number of responses as a major limitation in the discussion.

We excluded the data about tapering of drug doses because respondents gave a narrative explanation of their approach to this, and it was extremely difficult to pull out any common themes or ways of coding the data that gave a meaningful message. We have provided data on monitoring tests that were undertaken at follow-up visits and provide the total duration of treatment estimated by respondents. We also provide the full text of all questionnaire responses as a supplementary file if any reader wishes to review the exact responses about drug tapering.

Specific additional comments are found below:

24: Abstract: Define PCPs, BCCs, and IMHA at first use

These are now defined, line 25.

30: Technically it was also distributed to BCCs in ECC

We have adjusted to make this clearer, line 30.

37-38: Sentence intent could be clearer: All veterinarians made allowances for the weight of dogs in selecting a dosage. Most dosages were done on a mg/kg basis? The majority of clinicians used lower doses in larger dogs?

We have adjusted to make this clearer, line 37.

120-121: How many internists/ECC specialists do you approximate are in the UK? 69/8 is obviously not the whole group. Some reflection of what percentage of the diplomate pool this represents would be useful to acknowledge.

As noted above, we believe 8 was the total number of ECC diplomates in the UK at the time – this specialty has traditionally not been as developed as elsewhere. By reviewing the websites of all university and private specialist hospitals in the UK at the time of writing this response, we find 90 internal medicine diplomates in clinical practice, but this sector has expanded considerably in the last 5 years with many practices increasing in size or opening after being acquired by corporate bodies. We did not undertake the same type of survey at the time that the questionnaire was distributed so we cannot give an accurate percentage in this manuscript, but if there had been 90 practicing diplomates in 2016, we would have contacted 77% of them.

134-136: Good to know, but as mentioned above this data does not seem to appear in the manuscript.

We have added our data on tests completed at follow-up visits and the estimated duration of treatment (Fig 9, line 383 onwards), and the narrative responses about approaches to drug tapering can now be found in S2 Supplementary Information, column BJ.

141-142: Please perform statistical comparisons between groups.

We have added these comparisons – see extended methods section line 159 onwards.

148-149: I'm not sure that this needs to be included in the final manuscript, but since you had so much trouble recruiting specialists, I was wondering if the University specialists all came from RVC/Cambridge. May be useful to acknowledge how many universities/specialty practices that represented (if possible) since BCC numbers were so low and would be one reason for homogeneous responses from specialists if all responses came from a few facilities.

We do have this information: there was 1 respondent from the RVC and 1 from Cambridge (the authors did not complete the survey), which is very disappointing now that we realise it! 21 different universities/private practices are represented, and we have added this figure in line 177.

161-162: Include (PCP) (BCC) to define abbreviation in figures.

We have added these.

177-178: This sentence was not immediately clear to a non-UK reader, especially without context that the question was posed for patients with vs without a travel history; please clarify: "testing for vectorborne infectious agents absent a history of travel to a different country."

We have clarified the difference in testing for travelled and untravelled dogs in line 232, and expanded on this in the discussion line 493.

218-219: In legend for a, consider saying points represent values other than 2mg/kg

Actually, all individual points are plotted in this graph but there are so many responses of 2 mg/kg that they have all merged into a line.

238-239: The majority of individuals chose lower doses for the larger dog? Not all from data shown in D.

We agree, which is why we included this figure and specifically make the point in the text that the overall trend did not reflect the variability in individual responses. However, more than 50% of respondents did choose lower doses, so our statement is accurate.

250: Define BCC/PCP

We have added this.

260-265 & 269-270: This is the one place where I feel conclusions are overstated and statistics may not support what is portrayed as a difference between PCPs and BCCs. I also don't see this question in the text of the survey in supplementary info 1 so I assume they could only pick the top reason, while the question may have been better written as a ranking question since the decision to add a second drug is usually multifactorial, which is why I imagine all three options were chosen by a fairly large proportion of both groups (at least 25%). Question should be added to survey text (along with any others?) and I would emphasize that all three reasons were selected as important in both groups. The differences may turn out to be significant, but I think adding something to the effect that all represent important reasons to consider a second drug is valid.

We agree and have adjusted accordingly. Respondents could only write in as free text their reason (rather than ranking) and then responses were coded to produce these categories, line 345.

278: CsA is spelled both ciclosporin/cyclosporine in the text/figures

We have adjusted all to ciclosporin.

277-279: What is meant by this sentence? "...there has been a temporal trend for use of azathioprine, then ciclosporin (7), then mycophenolate mofetil (MMF) (8), and more recently leflunomide (9)." By specialists? GPs? As the most popular drug? That is unlikely true. This is the order the drugs were approved for use in humans so it is logical they came into more common use in that order, granted it took a bit for each to reach vet med. Clarify intent along with considering revising the following sentence.

We agree with the statement as made by the reviewer – these these drugs have entered veterinary usage in this order – and have revised accordingly, line 367.

275-278, 292-295, 312-216: Can this context be provided in intro or be moved to discussion? More discussion-like than what is typically found in results.

We agree in principle but feel this is important for the nature of this study, and we feel this is acceptable with the more flexible formatting for PLoS One.

320-321: "This decision was not similar between work settings (with 85/143, 59.4% of those in PCP and 9/21, 42.9% of BCC, choosing to vaccinate)" --> another place where I wondered if statistics would support the statement and a chi-square p-value is 0.15. Similar to above, the more important bottom line to me is that a large portion of both groups chose yes & no vs there was a difference between settings. I would consider those rates (60% vs 43%) fairly comparable given the paucity of data on the question... I do appreciate the study authors trying to gather data on current practices for this important question. Perhaps based on the discussion line 400 this was meant to be not "dissimilar"?

We apologise – this was an error, we meant to remove the word ‘not’ from a previous version. We have revised the sentence and added the statistical comparison, line 441.

340: Define endotype for the general reader

We have changed to subtype, line 464.

391: Minor correction of "frequently" rather than "frequency"

Corrected, line 538.

411: Limitations: Add small number of specialist responses.

We have added this in line 599 onwards with some other limitations of statistical testing in this study.

Comments on figures
Figure 1: Internal haematology and external haematology - suggest changing to in-house complete blood count and reference laboratory complete blood count.

Changed as suggested.

Figure 3: Found the vector-borne disease testing "if travel history" line confusing... also, in the survey, it offers never regardless of travel history, so may be clearer just to actually list the survey options. Open to other solutions.

We have added the actual survey options.

Figure 9b: Y axis- clarify % of respondents reporting any anticoagulant use, or something to that effect

Changed as suggested.

Reviewer #3: There is still scant information regarding the treatment and management of autoimmune hemolytic anemia in companion animal practice. This manuscript, while not earth-shattering, provides very valuable information to help advance the study of this condition. The manuscript is well written, and the data clearly presented. The only big question in this reviewers' mind is the differences in drug regimens between PCPs and BCCs and I agree with the authors that this warrants further investigation.

Thank you for your comments.
