# Supplementary material for: Cross-sectional study of approaches to diagnosis and management of dogs with immune-mediated haemolytic anaemia in primary care and referral veterinary practices in the United Kingdom
Source: PLoS One. 2021 Sep 20;16(9):e0257700. doi: 10.1371/journal.pone.0257700 (PMC8452064; doi:10.1371/journal.pone.0257700)
Supplement: Attachment — Submitted filename: Reviewer Comments R2.docx [file pone.0257700.s004.docx]

We are grateful to both reviewers again for their careful review of the manuscript and their assistance in its improvement.

Reviewer #1: Thank you for your thoughtful approach to addressing the reviewers comments and for the adjustments made to the manuscript in response; I believe that the manuscript is significantly improved.

Just a couple of minor remaining comments:
- the abbreviation ACVIM should be introduced the first time it is used (line 61), rather than line 73

Changed as indicated.

clarify "in the United Kingdom (UK)' on line 83, and then you can just use the abbreviation UK on line 91

Changed as suggested.

- Figure 5C, and 5D - consider adding adding "Prednisolone" prior to "dosage mg.kg per day" as the y axis label on these figures

Changed as suggested.

Reviewer #2: The manuscript is significantly improved after revision and the majority of my comments have been addressed. Statistical analysis strengthens the study's conclusions and is adequately described. A few minor inconsistencies and questions (line numbers referencing version with changes tracked):

- Line 34-42: Possible to add some P-values? Most statements are general, but the steroid dose being equal between groups and p<= 0.025 for lower steroid dose in larger dogs, for example? Anticoagulant use?

We have added these values for major comparisons.

- Add statistical descriptions to captions for figures to match others (Fig 4, line 249; fig 7, line 319; fig 10, line 376)

We have added the statistical descriptions for these figures.

- Line 226: Add reference for consensus statement

We have added the reference.

- Line 239-241: This section should include P-values from 4C

We have added the p values as suggested.

- Figure 5a has ns rather than the P-value

Changed to the actual p value (0.92)

- Line 305, 311-313, 403-404: Discrepancy in that 100% of BCCs say they use a second drug, but in 311-313 both groups often started with glucocorticoid and added a second drug if needed. 403-404 gives the impression BCCs always use two drugs. Please clarify.

Thank you – we have adjusted line 403 to reflect the correct meaning.

- Line 327-328: In line 176-177 you say the majority of respondents in both groups graduated between 2000-2015, so argument for why BCCs use the newer drugs more is unclear. Were they younger? Finished their training more recently?

We cannot answer this definitively because we did not ask about why respondents were using a particular combination of drugs and we did not ask for their age, but we would have 2 hypotheses:

- The years of initial graduation are similar overall between the two groups but BCCs will then have undergone their specialist training afterwards, so the length of time between formal education and this survey will have been shorter for BCCs.
- We suspect BCCs will be more aware of current trends in drug selection owing to their participation in listservs and other professional networks where, in our experience, these topics are often discussed.
